# Replication stress in *MLL*-rearrangements

**DOI:** 10.18632/oncoscience.281

**Published:** 2015-12-30

**Authors:** Michael Milyavsky, Boris Gole, Lisa Wiesmüller

**Affiliations:** Division of Gynecological Oncology, Department of Obstetrics and Gynecology, Ulm University, Ulm, Germany

**Keywords:** acute leukemia, aging, hematopoietic stem cells, replication fork stalling, secondary leukemia

Hematopoietic stem cells (HSC) are the only cells capable of self-renewal throughout the individual's lifetime and generate the whole spectrum of blood cells. Therefore genome aberrations in HSC can result in hematopoiesis failure or leukemic transformation. Chromosomal translocations, inversions, amplifications and complex rearrangements at the 11q human genomic locus encoding *mixed lineage leukemia gene* (*MLL*) are the hallmark of several blood malignancies including infant, therapy-induced, donor - and *de novo* leukemias. The vast majority of these 11q aberrations fall within a 7.3kb *MLL* breakpoint cluster region (*MLL*bcr) with a particular hotspot at the intron11-exon12 boundary [1]. Intriguingly, a large variety of genotoxic, cytotoxic and biological stimuli were connected with *MLL*bcr breakage pointing to the existence of several DNA cleavage and repair mechanisms acting at this locus [1, 2]. From the broad spectrum of stimuli triggering cleavage in concert with diverse mutagenic outcomes at the locus it is tempting to seek for a common molecular process engaged.

**Figure 1:**
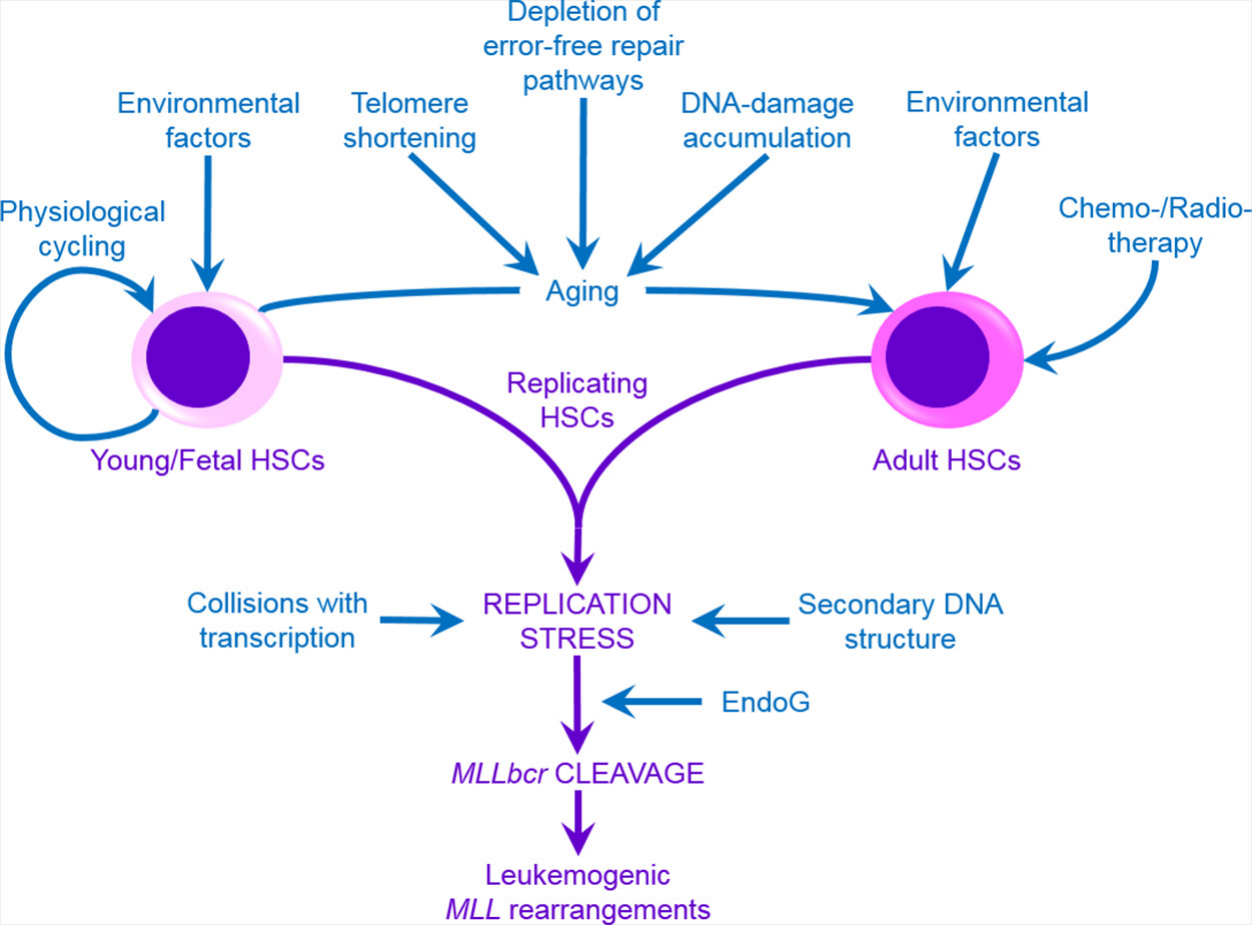
MLL rearrangements can result from failure to correctly resolve replication stress in HSC Multiple exogenous and endogenous factors can trigger excessive HSC cycling and/or provoke replication blockage. *MLL*bcr is a frequently targeted substrate of replication stress-induced cleavage such as by Endonuclease G (EndoG). Failure to bypass stalled replication forks and to repair collapsed forks in an error-free fashion can result in leukemogenic *MLL* aberrations.

Based on our and others’ experimental evidences, we postulate that replication stress in HSC can be responsible for *MLL* rearrangements (Figure 1). Thus, our data revealed *MLL*bcr breakage upon mere replication blockage via DNA polymerase inhibition or upon exposure to the nucleoside analog 5-fluorouracil [2]. Induction of HSC's specific replication stress can be linked to many agents and conditions implicated in *MLL* leukemias. Normally, quiescence of HSC with only rare replication cycles accompanied by low metabolic activity and ROS levels contributes to minimize the mutational load under homeostatic conditions [3, 4]. In contrast, forcing HSC into excessive cycling by chronic stimulation with physiological triggers mimicking inflammation, bleeding or cytopenia provokes a robust DDR that drives both HSC death and mutagenesis of the survivors. Thus, Walter *et al.* [4] detected DDR markers associated with replication fork stalling and collapse such as DNA breaks and nuclear γ H2AX, 53BP1 and FANCD2 foci upon enforced HSC exit from quiescence. Transplantation induces rapid cycling of normally dormant HSC that can be exacerbated by donor immunosuppression, damaged microenvironment and altered cytokine profile. Signs of endogenous DNA damage upon serial transplantation of HSC are well documented in both humans and mice with evidence for altered DNA replication dynamics, chromosome gaps and breaks indicative of replication stress [3, 5]. We suggest that exhaustion or failure of replication stress-associated high fidelity repair pathways under transplantation challenge can be implicated in donor cell-derived acute leukemia with *MLL* translocations in patients who received HSC transplant [6]. Given the fact that replication stress in HSC is associated with aging [3] one can hypothesize that *MLL* rearrangements, particularly amplifications often associated with complex rearrangements [7], observed in *de novo* AML in the elderly are the consequence of replication stress associated DNA repair failures.

Induction of replication stress in HSC can also be linked to agents and conditions associated with common solid cancer therapies. Indeed, topoisomerase II inhibitors and cytostatics with a different mode-of-action such as alkylating agents or 5-fluorouracil [1, 8] but all implicated in the etiology of therapy-induced leukemia or *MLL* rearrangements can recruit dormant HSC into the replication cycle as a result of chemotherapy-associated cytopenia. Moreover, fetal HSC which are the infant leukemia cell-of-origin are highly cycling populations and thus collision of replication forks with lesions can cause overwhelming replicative stress. Altogether, replication stress may represent the integrating signal in HSC following genotoxic exposure of the fetus and of patients undergoing radio- or chemotherapy, in HSC undergoing excessive self-renewal in the fetus or upon transplantation as well as in HSC from aged individuals suffering from the exhaustion of replication factors [3]. The extraordinary susceptibility of the *MLL*bcr to replication stress-induced breakage may stem from its secondary structure resulting in the collision of transcription and replication machineries recruiting nucleases such as Endonuclease G in decondensed chromatin [1](Figure 1).

To summarize, replication stress response plays a key role in regulating HSC function. We anticipate that deeper understanding of associated molecular mechanisms responsible for *MLL*bcr cleavage and subsequent repair in HSC can hold the key for future chemoprevention and anti-aging modalities.
