# Erratum: Replication stress in MLL-rearrangements

**DOI:** 10.18632/oncoscience.303

**Published:** 2016-04-19

**Authors:** Michael Milyavsky, Boris Gole, Lisa Wiesmüller

***Oncoscience. 2015 Dec 30;2(12):938-9***

**PMCID**: PMC4675780**PMID**: 26909360

**DOI**: 10.18632/oncoscience.281

In this Editorial, corresponding authorship was listed incorrectly. Authors Michael Milyavsky and Lisa Wiesmüller shared corresponding authorship of this paper. It is corrected as follows:

Corrected correspondence information:

**Michael Milyavsky**: Department of Pathology, Sackler Faculty of Medicine, Tel-Aviv University, Tel-Aviv, Israel

**Correspondence**: Michael Milyavsky, mmilyavsky@post.tau.ac.il

**Lisa Wiesmüller**: Division of Gynecological Oncology, Department of Obstetrics and Gynecology, Ulm University, Ulm, Germany

**Correspondence**: Lisa Wiesmüller, lisa.wiesmueller@uni-ulm.de

